# The Integration of Flexible Assertive Community Treatment with District Social Support Service Teams for Patients with Severe Mental Illness in the Netherlands: A Qualitative Analysis of Team Members’ Experiences

**DOI:** 10.5334/ijic.9794

**Published:** 2026-09-08

**Authors:** Anne Kleijburg, Christien Muusse, Ben Wijnen, Joran Lokkerbol, Pim Candel, S. Kor Spoelstra, E. C. Marjolijne de Jong, Silvia Evers, Hans Kroon

**Affiliations:** 1 Department of Health Services Research, CAPHRI Care and Public Health Research Institute, Maastricht University, Maastricht, The Netherlands; 2 Centre for Economic Evaluations, Trimbos Institute, Netherlands Institute of Mental Health and Addiction, Utrecht, The Netherlands; 3 Department of Reintegration and Community Care, Trimbos Institute, Netherlands Institute of Mental Health and Addiction, Utrecht, The Netherlands; 4 EHDK, an integrated healthcare consultancy agency, Amsterdam, The Netherlands; 5 Department of Addiction, Addiction Care North Netherlands, Groningen, The Netherlands; 6 Department of Healthcare, NHL Stenden University of Applied Sciences, Leeuwarden, The Netherlands; 7 GGZ Friesland Institution for Mental Healthcare, Leeuwarden, The Netherlands; 8 Department of Social and Behavioural Sciences, Tranzo Scientific Center for Care and Welfare, Tilburg University, Tilburg, The Netherlands

**Keywords:** integrated care, severe mental illness, flexible assertive community treatment, mental healthcare, social services, implementation

## Abstract

**Introduction:**

To promote recovery of individuals with severe mental illness and reduce fragmentation of care, network partners collaborated to develop a cross-domain, multi-agency approach by integrating Flexible Assertive Community Treatment (FACT) teams with municipal District Social Services (DSS), referred to as FACT+DSS teams.

**Aim:**

To describe how team members experienced the implementation of the FACT+DSS teams by team members, including perceived effects on various themes such as service delivery, network collaboration, the target population, and facilitators and barriers to implementation.

**Methods:**

A qualitative study using semi-structured interviews (N = 15) was conducted to explore the experiences of team members in two new FACT+DSS teams in the Netherlands using thematic analysis.

**Results:**

The FACT+DSS approach was experienced to improve service access, continuity of care, case-finding and client enrolment (care-avoiders), time efficiency for team members, and strengthen network collaboration. Identified barriers and facilitators underlined the importance of strong leadership, ownership, equality, direct relationships, and professional expertise.

**Conclusion:**

Participants experienced the FACT+DSS approach as a highly positive development, improving network collaboration and recovery-oriented care for clients. Our findings may contribute to the development and implementation of FACT (and related practices) as an integrated multi-agency approach.

## Introduction

Severe mental illness (SMI) is defined as any mental disorder lasting at least two years, substantially interfering with multiple life domains, including physical and mental wellbeing, social functioning, work, education, housing, and finances [1]. To support recovery of individuals with SMI, several dimensions can be distinguished, including clinical recovery (relief of psychiatric symptoms), functional recovery (re-establishing social and executive functions, and self-regulation), and personal recovery (re-establishing positive personal identity) [2,3,4]. Some frameworks additionally distinguish societal recovery (meaningful participation in society), and existential components (meaning and identity). Therefore, recovery-oriented care requires a broad perspective, addressing all different life domains in addition to treatment of disease symptomatology [2,3,4,5].

Since the end of the 20^th^ century, mental healthcare services (MHC) and addiction care (AC) in the Netherlands have undergone phases of deinstitutionalisation, aimed at improving the quality of health services, and stimulating social inclusion, participation, and quality of life. To achieve this, community-based care models were increasingly implemented, evolving iteratively from case management to Assertive Community Treatment (ACT), and subsequently into the Dutch Flexible Assertive Community Treatment (FACT) model [6]. Core principles of FACT include using a multidisciplinary team approach to provide and coordinate recovery-oriented, intensive outreaching care, integrating ambulatory treatment and supportive social care, that can be up or downscaled according to clients’ needs [7]. To promote quality of care delivered using FACT, since 2008 the Centre for Certification of ACT and FACT (CCAF) has provided fidelity scales, team workbooks, and audits. As the organization of care changed significantly over time and between local contexts, this translated to large, contextual variations in needs for FACT to delivering integrated care. To reflect these changes, in 2017 an update to the original FACT scale (2008) was published, supporting teams to organize integrated care in line with context-specific needs, with additional revisions in 2022 (FACTs 2017-R) [8]. Past and present developments of the FACT model have been outlined elsewhere [6,9].

The Dutch government introduced new reforms in 2015 increasing extramuralisation and decentralisation, aimed at stimulating quality, societal participation, and cost reduction [10]. Reforms shifted the responsibility of organizing social services and long-term supported living to municipalities, fragmenting services previously delivered integrated from within FACT across agencies. This included financing of care for individuals with SMI now being distributed across: 1) the Health Insurance Act, covering all curative health care, including FACT, 2) the Long-term Care Act (Wlz, 2015), covering long-term psychiatric hospitalization (and from 2021 also long-term sheltered housing), and 3) the Social Support Act (WMO, 2015), covering municipal social. Accordingly, financial responsibility is managed on three levels by: 1) health insurers (nation-wide), 2) regional care offices, and 3) municipalities.

From 2015 onwards, municipalities introduced a wide, but highly variable range of support services, often accessible via District Social Service Teams (DSSTs) or municipal counters. Although working methods may vary regionally, DSSTs often operate community-based where caseworkers supporting citizens with services targeting e.g. domestic functioning, housing, work, daytime activities, income or social benefits, and social/family relationships. Upon delivering recovery-oriented social services towards multiple life domains, this means the responsibility of caring for individuals with SMI in the community is shared between FACT and the DSSTs, requiring close collaboration and deliberate service alignment [8]. When lacking, this may introduce inefficient redistributions of responsibility. For example, proactive assertive outreach (e.g. for care-avoiders with SMI) is a core intervention of FACT, however, can be limited when individuals refuse treatment and involuntary treatment is not indicated [11]. When ongoing mandate extends beyond the mandate of FACT, other actors must assume responsibility for individuals who avoid care or cause community disturbance. Some regions organised continued outreach and case-finding through separate, specialized teams (Dutch: *Critical-Time Intervention or bemoeizorg/OGGZ-teams*), whereas in municipalities without dedicated resources this responsibility was often transferred to DSSTs despite limited psychiatric expertise [11].

Upon fragmentation, FACT had to increase its coordinating role to deliver integrated services. Efficient and consistent collaboration with cross-domain partners is essential, both to support recovery-oriented services and to ensure sustainable use of organizational and financial resources (i.e. coordination time is only partially reimbursed and reduces direct patient time). However, the required level of network collaboration has proven often too structurally challenging to sustain, with reasons including financial constraints, lacking communication, (privacy) laws and regulations (e.g. limiting information exchange), complex care pathways, application procedures, and misaligned organizational incentives and collaborative visions [10]. Collectively, this contributed to FACT becoming increasingly restricted to providing specialised MHC, rather than adopting a proactive community approach e.g. by preventing escalation with outreaching care without a formal diagnosis, as illustrated in detail by Muusse et al. (2021) [12].

Over the past decade, the need for developing cross-domain, multi-agency approaches to FACT specific to the local context has been increasingly emphasized [8,13,14,15,16]. This way, challenges imposed by fragmentation can be reduced, promoting recovery outcomes for clients and (cost-)efficient use of resources (e.g. labour capacity) [13,15]. The FACTs 2017(-R) has been developed in support of such approaches, explicitly accommodating context-specific variations to integration. Examples include (but are not limited to) variation in 1) structural levels e.g. on a team-level (delivering integrated services from within a team) or on a network-level (delivering integrated services by aligning these between providers), and 2) the degree of integration ranging from low (e.g. limited collaboration efforts) to complete (e.g. full merger of practices, systems and resources), and 3) the scope of integration, reflected by the number and type of partners integrated with [8]. International experiences demonstrated that FACT can be adapted effectively using deliberate, context-specific approaches. For example, in Norway fragmentation was reduced upon team-level integration of FACT using a binding collaboration (i.e. mandated) between primary (municipal responsibility) and specialist (state responsibility) care, integrating nearly all services within the team [16,17,18,19]. Similarly, Dutch initiatives using different variations to integration with FACT have also been implemented, as discussed by Westen et al. (2025) [6].

### The Frisian FACT+DSS approach: integrating FACT and District Social services

This study examines a local multi-agency initiative developed in two municipalities in the Dutch province of Friesland, aiming to optimize cross-domain, recovery-oriented care by improving access, personalization, and efficient coordination of services, tailored to each individual needs. A detailed description of the initiative (including team composition, setting, working methods) is provided in the study protocol by Kleijburg *et al*. (2022) [20].

In short, local network stakeholders, consisting of a MHC provider, an AC provider, a health insurer, two municipalities, and the employment insurance agency (EIA), developed the multi-agency FACT+DSS approach. This approach aimed to increase integration of services on a team-level, and on a network-level by improving collaboration with stakeholders, whilst organizational mandate and financing of resources remaining based at each respective stakeholder. In this study two FACT+DSS teams were implemented, consisting of 1) the former FACT team from the MHC provider, 2) two or three social workers from the municipal DSST (team-level integration), 3) an EIA occupational consultant for employment reintegration services (close collaboration with remote access), and 4) at least two FACT team members specialized in addiction from the local AC-provider. Here, in one FACT+DSS team team-level integration had already been established. In the second FACT+DSS team, organizational agreements resulted in a remote, close collaboration with FACT+DSS to facilitate consulting access, including presence of AC-expertise in the FACT+DSS team in line with the FACTs 2017(-R)). Daily practices of all FACT+DSS team members (including DSS and AC) are in line FACTs 2017(-R), consisting of joined daily team processes (FACT-board meetings, house visits, co-location) and shared caseload management (case-managing, shared documentation). The FACT+DSS approach requires each client to receive an integrated intake which, in addition to the assigned case-manager(s) for MHC and/or AC, must include a DSS-social worker. Next, a personal treatment plan is created including all indicated MHC, AC, and/or DSS supportive of recovery as early as possible.

For project management, stakeholders formed a steering group, supported by a team of project consultants, working with organizational advisors, and lawyers, to prepare implementation and navigate shared financial responsibilities, privacy policies, information sharing, operational agreements, and other formal responsibilities between partners.

### Aim

This current study aims to describe how team members, client representatives, and a project leader experienced the implementation of the FACT+DSS teams, as well as perceived effects on various themes including service delivery, network collaboration, the target population, and facilitators and barriers to implementation.

## Methods

### Study context & design

This implementation process evaluation of two integrated FACT+DSS teams was part of a 2-year longitudinal, quasi-experimental study with an implementation period spanning from April 2020 to April 2022. Teams were implemented in two Dutch-Frisian municipalities, one being more urban (Leeuwarden, 526 inhabitants per KM2) and the other being more rural (Súdwest-Fryslân, 172 inhabitants per KM2).

Data was collected throughout the study period to inform process evaluation questions based on the framework by Saunders *et al*. for health promotion programs [21]. This framework consists of six elements; fidelity, dose delivered, dose received, reach, recruitment, and context, and guided both the collection of data (e.g. to structure interview guides) as well as the initial coding structure during data analysis.

Additional methodological details are reported in Supplementary Table S1 using the Consolidated criteria for reporting qualitative research (COREQ) [22].

### Data collection

#### Interviews

Interviews provided the main data source informing participant experiences and perceptions. Towards the end of the implementation phase, 12 semi-structured interviews were performed (3 interviews included two participants), totalling to 15 participants by (criterion-based) purposive sampling (Table S1). Participants included 12 FACT+DSS team members, a project manager, and 2 client representatives (former FACT-clients) from the MHC-provider’s council (participant details in Supplementary table S2). Interview themes were structured using elements from the Saunders framework and aimed to gain insights on themes such as case mix, multidisciplinary involvement, network collaboration, use of social services, care avoiders, and implementation barriers and facilitators (overview provided in S3). Interviews were performed at team office locations or online, with a maximum of two participants per interview when preferred. All interviews (45–90 minutes) were performed in Dutch, audiotaped, and transcribed verbatim. Included quotations were translated verbatim by the first author.

#### Documents

Additionally, a variety of available documents were collected for supportive insights, including 1) steering group meeting minutes and early-stage team-monitoring forms, to keep track of developments and/or challenges throughout implementation, as well as insights regarding e.g. perspectives and attitudes of steering group members, and 2) supportive implementation materials provided to team members, e.g. handbooks and illustrative case-studies, to inform reflections on their usefulness. Furthermore, researcher notes collected by the first author (A.K.) during on-site walkalongs, joining the FACT+DSS teams in their daily activities, were also included.

### Data analysis

Analysis of transcribed interviews and collected documents was conducted using the coding software MAXQDA v.2022. Data analysis was performed using a two-step approach. First, upon familiarization of the data, codes were assigned both inductively and deductively, with identified themes structured based on the six components of the Saunders framework and interview structure. Second, to narrow the focus towards “what’s new”, a second thematic analysis further condensed initial themes, including a reorganization of the associated codes. The coding and identification of recurring themes was performed by the first (A.K.) and second author (C.M.) to ensure consistency. Furthermore, identified final themes were discussed and reviewed by all co-authors, of which some were directly involved with the project implementation (management and/or clinician), as a final validation step of interpretation.

### Ethics and consent

This study was approved by the Medical Ethics Review Committee of the University Hospital Maastricht and Maastricht University (application no. 2021-2868) as part of the complete study protocol. The purpose of the study was explained to all interview participants, after which participation was voluntary. Despite anonymisation, the limited number of participants can cause identification amongst colleagues or stakeholders. Therefore, participants were asked to provide verbal consent for publishing quotations.

## Results

Data analysis revealed a series of major themes relating to key experiences and outcomes during implementation of the integrated FACT+DSS approach. Key findings presented in Table 1 have been organized by four themes describing “what’s new” in terms of 1) providing care-related activities, 2) collaboration with the (local) network, 3) the client target population, and 4) key facilitators and barriers experienced throughout the implementation process.

**Table 1:** Overview of final theme selection.


THEME	WHAT’S NEW IN TERMS OF:	KEY FINDINGS

**1**	Providing care-related activities?	Integrated intake & treatment plans Direct application to support service indications Use of alternative services promoting scaling down or discharge Improved awareness on distinction between treatment vs supports Reduced administrative burden & improved time-efficiency

**2**	Collaboration with the (local) network?	Improved communication and understanding

**3**	The client target population?	Faster recognition of target population Social worker’s key role in the enrolment of care-avoiders into FACT

**4**	What factors were experienced as important facilitators and barriers during the implementation process?	*Facilitators:* Strong leadership and vision Importance of ownership & culture change Reducing culture differences & emphasizing equal importance Establishing and maintaining personal/direct relationships between care professionals *Barriers:* Impact of external stressors Lack of experience of social workers in social domain


### 1. What’s new in terms of providing care-related activities?

#### Integrated intake & treatment plan

The FACT+DSS approach requires each client to receive an integrated intake and integrated treatment plan, including expertise of each domain (MHC, AC, DSS) when needed, and align all services throughout treatment phases. Before implementation, team members recognized often lacking awareness and knowledge regarding the value and availability of DSS. Upon integration, team members described learning a lot from each other’s expertise throughout cases, gaining transdisciplinary knowledge and becoming increasingly aware over time of the added value of integration for clients and its contribution to recovery. For example, by reducing the burden of external stressors and non-disease related factors (e.g. housing, financing or childcare), or by promoting a sense of autonomy and fulfilment in life (e.g. employment or daily routines).

*Nurse practitioner: “Recently, I ended treatment with a woman who had dual diagnosis problems, […]. When doing this I always ask people what has been helpful in their recovery… and to my great surprise she responded that we arranged childcare for her children. Arranging day-care for children is not something we would even consider including as part of a treatment plan in the past… But […] she was finally able to let go a little of her mother role and focus on the care that she actually needed very much.”*

However, team members acknowledged that during high-workload periods sometimes invitations for social workers to the integrated intake were forgotten, recognizing clients potentially missing opportunities to receive support services targeting other domains.

Another important benefit of integration with DSS social workers is that their service delivery can be directed towards all citizens in the municipality, meaning services can also be more easily extended to the client’s system (e.g. children, family, or informal caregivers) to provide them with DSS when needed, and also supporting them in their informal care or family role.

#### Facilitating application for social support service indications

Before integration, clients would often have to apply for an DSS-indication themselves with the municipality or DSST, however, it was recognized that this was complex and clients would often not (successfully) apply.

*Psychiatrist: “I think that everybody should work like this. Because especially when you have this client population, they have problems with so many aspects: social, healthcare, relationship problems, financial problems, you name it. […] Now we can just organize it instead of telling our very sick clients “I think you need this service but we can only help you with healthcare, so if you see an opportunity you should go there to apply for it yourself”. I think that’s really the opposite of how it should be.”*

This complexity and limited access was also recognized by the client representatives. For example, when approaching discharge, they were often advised to seek social support services, as FACT teams were often limited in their capacity and ability arrange this. This could contribute to feelings of frustration or fear of consequences when not receiving support post discharge.

*Client representative 1: If you come out of treatment then… apart from the fact that the problems may come back, you also have to deal with very practical things, such as contacts with the municipality or a health insurer or yes, you name it. The average citizen would already have their hands full with that, let alone if you’re not feeling well or your living situation is very difficult.”*

In some cases, FACT team members could support clients with their application procedures, which was often time-consuming and, also from lacking knowledge on the social domain and poor communication, could still result in rejected applications. Now, DSS-social workers can provide immediate advise during team meetings to organize the right services, or contribute service suggestions services when recognizing opportunities during discussion.

*Social psychiatric nurse: “I think it’s great that we can organize those things from the start with each other now. I also had clients with whom the social worker was remote. […] It’s much more distant, so sometimes it doesn’t go as well.”*

#### Use of alternative services promotes scaling down or discharge

Team-level integration has also improved recognition of opportunities for scaling down FACT care and discharge of clients by providing alternatives. Team members explained that the knowledge of DSS-social workers regarding the (often wide) variety of locally available support services (i.e. for coaching/guidance, daycare activities), allowed social workers to propose appropriate alternatives to FACT when evaluating clients cases. For example, for clients who no longer require treatment by FACT but would benefit from continued support-based contacts and monitoring. Furthermore, when support services are initiated during earlier stages of care, clients feel more prepared for discharge facilitating a smoother transition and sense of security. This was also recognized by the client representatives, as clients often experienced discharge as abrupt and feared for relapses.

*Nurse practitioner: “I also think that we utilize the right support service earlier, so that you can scale down FACT care itself, the more expensive treatments…. And because we deploy it earlier, it’s not like at the time we’re talking about discharge, it still has to be organized. […] So that means that by the time we start to scale down or finish, the people are already known or more familiar.”*

To facilitate smooth transitions for clients upon discharge to alternative providers, FACT+DSS teams introduced communication plans providing a ‘warm transfer’, informing new support providers on a client’s history, and provide signalling plans and contact information in case of alarming situations. Additionally, these warm transfers promote approachability and awareness of expertise, allowing for faster responses in (potential) crisis situations for clients, improved continuity of care, and ultimately recovery.

*Psychiatrist: ”Some people also deserve to get out of care. Some people have been very sick and they’re fine, they’re stable, but they still need some support, […]. It’s much healthier to just do that from the community…. With that understanding, the moment things don’t go well yes, within one to two weeks you are back in care with us. And that, I think, is also much better than it was.”*

#### Impact of treatment vs support on discharge

During implementation, some clients were recognized to no longer need care from FACT, but did still require continued support services from the FACT+DSS social worker. This raised questions about the teams’ role in providing treatment versus support services, and when discharge from FACT+DSS (including transfer to alternative care) would be appropriate:

*Walkalong notes: “The limits of the terms “Support” and “Treatment” are not always clear. The counselling provided during support services also has treatment aspects and thus growth/change opportunities, and similarly every treatment has counselling/support aspects for maintaining quality of life and structure. Where exactly do you draw the line and at which moment?” (from a conversation with a social psychiatric nurse)*

Allowing clients to remain in the FACT+DSS team whilst only needing support services (without FACT care), would ultimately impact the teams’ caseload size and the inclusion/exclusion criteria specifying its target population (or case-mix). Alternatively, arguments could also be made to continue support services by FACT+DSS, given continuity of care and trust-based relationships are both considered key values of the FACT+DSS approach. Ultimately, stakeholders decided to approach these situations with personalization and flexibility, however, should be limited to exceptional cases or for limited periods of time, until other appropriate services can be organized.

#### Improved time-efficiency & administrative burden

Stakeholders had aimed for integration to reduce the administrative burden by simplifying the registration system, but this was recognized to provide only minor relief. Nevertheless, team members did describe other mechanisms contributing to a reduced sense of administrative burden and improved time-efficiency. For example, direct access to services of the DSST or AC has significantly reduced the amount of time spent on time-consuming tasks including application procedures, consultations, or meetings to coordinate services with other providers. As a result, saved administrative time can be spent on activities relating to their professional expertise instead (e.g. direct patient care).

*Social psychiatric nurse: ”I really think that care is more personalized now. The speed at which things get organized, I find really remarkable. Also, what is really nice is that everyone who works in this team can now do more of what they’re good at. For example, a practitioner who is very good at providing cognitive behavioural therapy no longer has to deal with municipal financial support, because we have people with that expertise now.”*

### 2. What’s new in terms of collaboration with the (local) network?

#### Improved communication and understanding

Upon integration, team members identified two important developments which positively influenced the local network: 1) shorter communicative lines between (cross-domain) actors (e.g. service providers, local police, housing association, municipal regulators of public order and safety), and 2) an improved understanding of each other’s roles and restrictions. Prior to implementation, the lack of communication and understanding between organizations frequently caused mutual frustration and blame. For examples, regarding differences in restrictions imposed by patient confidentiality, privacy laws, and/or the inability of FACT to act when treatment is not voluntary, e.g. when individuals with (suspected) SMI cause community disturbances. In these situations, the DSST and the local police were required to take responsibility, despite lacking necessary psychiatric expertise and resources. Now, working within a single team stimulated ongoing conversation between parties at the team and management level, creating space to understand each other’s considerations and restrictions, and more importantly, find solutions to provide individuals with the right care. Furthermore, wider network communication was largely facilitated by existing relationships of the DSS-social workers, where positive experiences over time stimulated parties to reach out more frequently. In one of the FACT+DSS teams, this resulted in the implementation of a consultation phone line, including the possibility to join team meetings for consulting on client cases.

*Nurse: “We are regularly contacted by different parties now, such as a local police agent or DSST social workers. To discuss any problems […], and to see if we can take a look or advise them. For example, recently […] people from the housing association had looked inside an apartment from a crane and saw that the apartment was completely trashed. After that they looked into it and found that there had also been reports of nuisance in the past. So then we were contacted by the housing association and local police to take a look, and we made contact with this person and offered to clean his house. It took some time because he was very traumatized, but the result is that he now [..] wants to come into treatment. Those are very nice collaborations.”*

### 3. What’s new in terms of the client target population?

#### Faster recognition of target population

During the interviews, the possible impact of integration between FACT and DSS was considered on potential changes in (the definition of) the teams’ target population (i.e. impacted by its diversification in provided services). Here, FACT+DSS team members describe not experiencing differences in definition of their target population, as this continues to consist of individuals with SMI. However, they recognized that the improved network communication did have a positive impact on the identification of individuals belonging to the target population within the catchment area, accelerating FACT involvement and enrolment into care.

*Social psychiatric nurse: “I think the target group has not changed but we can find them better. I think in the past, a lot of DSST members have been caring for clients by themselves for a very long time, perhaps together with a general practitioner, […] we have often said in the past, saying: ‘those care-avoiding individuals, we should really be in the picture earlier’.”*

#### Social worker’s role in the enrolment of care-avoiders into FACT

Convincing care-avoiders to accept (FACT) treatment was often experienced as a highly complex process, often due to their lack of interest or trust. Upon integration, FACT+DSS team members recognized that the DSS-social workers could play a crucial role in this process by 1) increasing recognition of care-avoiders, and 2) establishing trust-based relationships necessary for convincing care-avoiders to accept treatment. Because social workers are generally perceived as less threatening, they could take the lead by reaching out first, offering DSS providing them direct benefits (e.g. house cleaning or financial benefits) to gain trust, and enrol clients into the FACT+DSS caseload. Once trust was established, other FACT+DSS members could slowly be introduced to discuss treatment options.

### 4. What factors were experienced as important facilitators and barriers for (maintaining) integration?

#### Facilitator: Collaborative mindset and commitment on all levels

On both the team and administrative level, a collaborative mindset and a high level of commitment were essential for the development and implementation of the FACT+DSS approach. In practice, this means having an open mind, being curious towards each other, and committing to understand and learn from each other’s perspectives, motivations, and languages. When achieving this, this created space to find creative and innovative solutions to common problems, which may not have seemed possible before i.e. relating to existing restrictions in the current health system.

#### Facilitators: Strong leadership

A second important facilitator was the presence of strong leadership. The team managers and coaches were described as crucial for strengthening relationships and mutual understanding between the managers of FACT+DSS partner organizations by remaining in frequent contact with each other. Furthermore, the coach motivated team members using repeated activation and engagement throughout implementation, motivating both old and new team members to remain aware of the reasoning behind working with the integrated approach, and change their behaviours accordingly. Here, team members recognized that sometimes, but especially in periods of high workload (e.g. high caseload, staff shortage, training new members, added responsibilities), awareness for actively collaborating and integrating treatment was more likely to be forgotten, stressing the relevance of strong leadership to sustain service integration over time.

#### Facilitator: Creating ownership

A third facilitator was the importance of creating ownership to strengthen the collaborative mindset. Here, the additional, dedicated team meetings that were organized during the implementation phase were described as especially important. These meetings focused on e.g. identifying collaboration opportunities by discussing case studies and how these could be improved using a cross-domain approach, and on creating ownership through a deeper understanding of the rationale behind the integrated approach. Importantly, since these meetings were mostly organized during the first year of implementation, a few new team members (not present at start) expressed sometimes lacking the same understanding and felt that repeating these meetings could enhance their comprehension.

#### Facilitator: Reducing culture differences & emphasizing equal importance

A fourth facilitator emphasizes the importance of recognizing differences in organizational cultures and its impact on collaboration and integration. Prior to implementation of the FACT+DSS approach, stakeholder project leaders were already very aware of the importance of establishing a shared vision and equal importance amongst team members. This awareness stemmed from past collaboration experiences during which the MHC stakeholder was perceived as more hierarchical, rigid, and dominant, requiring professionals of collaborating partners to conform their procedures and rules, which created negative sentiments (i.e. unappreciation, inequality). To prevent similar sentiments, integration was intentionally narrated as the formation of a completely new team, rather than social workers or addiction-care specialists joining the existing FACT team. Furthermore, team managers and coaches played an important role in reducing the existing culture differences by repeatedly emphasizing the equal importance of all specializations and domains.

“Coach/psychiatrist: “Something tricky here is, that before you know it, people can get the perception that *the integration* is just more people who can go and do some of the work for *MHC-FACT*. […] So we introduced the theme of being equal to each other. Everyone still has their own work, but that we keep looking for; how can we, actually cross these barriers, really letting go and work together? And that actually worked out very well.”

#### Facilitator: Establishing and maintaining personal/direct relationships between care professionals

The fifth facilitator concerns the ability to establish and maintain personal and direct relationships between care professionals, and how this may contribute to continuity of care. Prior to integration, communication between domains was available on consultation basis, however, in practice, professionals describe often having experienced a sense of reluctancy to reach out e.g. due to being too time-consuming, or their consultation request being too uncertain or vague. This can result in missed opportunities for providing the “right”, i.e. the most appropriate and effective, course of action. Given that the ability to recognize when and how to provide the “right” care over time is essential for continuity of care, personal relationships and physical proximity (i.e. co-locating) between cross-domain professionals reduce such barriers. This is illustrated by the quote below relating to the experience of a social worker and the importance of being able to quickly communicate with the psychiatrist about the plan of action for a complex client case.

*Social worker: “Before I never had direct contact with the psychiatrist and now I do, so you notice that you can align approaches with each other much better when you prepare for major interventions.”*

An example involves managing the clean-up of a trashed house belonging to a care-avoiding women who hasn’t been outside for three quarters of a year.

*Social worker: “There is also the question of how far can we go? Because I am the one who directs this action. I have the lines of communication with the housing corporation and the police, that’s how I use my expertise. But in the meantime, this lady has to be doing well psychiatrically, so to speak. So, whether that pace I have in mind, whether I can follow that, that is what I have to know from the psychiatrist. […] Normally, you would have to e-mail around first, it would take a lot of time before you have an answer. But now, because you’re in the same corridor you just walk by, ask your question, and then I can move on. […] You see that you can use the network you have created, a widened network, much better.”*

#### Barrier: Impact of external stressors

Throughout implementation, several external stressors, events, or other factors were identified that negatively impacted the (maintained) implementation of the integrated approach. Frequently mentioned examples are 1) the Covid-19 pandemic, which begun just before implementation in 2020; 2) reduced staff capacity, due to absenteeism from sickness, staff shortages, unfilled vacancies, and periods of training for new team members; 3) national and/or local changes in policies and/or laws resulting in additional workload, e.g. implementation of a new registration and financing system for MHC in the Health Insurance Act, the opening of the Long-term Care Act in 2021 for clients with SMI, and local stakeholder reorganizations; and 4) other complicating factors that required resolution at higher management or project leadership level (e.g. problems with Electronic Health Record systems, or lack of policy on privacy-related problems). Team members described that these stressors would often introduce extended periods of additional (time) pressure on the team, e.g. by increasing caseload size or adding tasks to the workload of team members, and acknowledged that this compromised their ability to collaboration effectively.

#### Barrier: Lack of experience of social workers in social domain

Finally, it was recognized that an important barrier to the success of the integrated approach was related to the social workers’ level of experience and knowledge regarding the local social domain and its available services, contacts, and procedures. In case of lesser experience, team members described being less inclined to approach the social worker for help as their added value was not as clearly recognized, compromising the added value of the integrated approach.

*Psychiatrist: “I think it’s unfortunate … That people are hired specifically for this position who don’t know the municipality well. Because with that you lose added value. The advantage is in having someone who is well embedded in the municipality, so that you can provide all the care that the municipality offers… […]. So yes, if someone comes here blank, it takes both a lot of work to get to know the municipality and also a lot of work to get to know FACT.”*

## Discussion

### General

In this paper, we aimed to describe the experiences and perceived effects of implementing FACT+DSS teams, a cross-domain initiative integrating municipal social workers into FACT and strengthening collaboration with network partners, including addiction care. In line with its aims [20], findings suggest that the FACT+DSS approach supports recovery-oriented care for clients by improving access to social services, continuity of care, and cross-domain treatment planning. DSS-social workers were described as playing a crucial role in facilitating client access to municipal services, client enrolment, building trust-based relationships with clients, and strengthening network collaboration. Overall, these findings support that context-specific multi-agency collaboration can enhance recovery-oriented care within fragmented systems for individuals with SMI.

### Reflection in the context of the international evidence

Over the past decades, the FACT model has expanded internationally to countries including Norway [16,18,19], Sweden [23], Denmark [24], and Lille [25]. Despite variations in national health systems, fragmentation and insufficient network collaboration have resulted in comparable challenges, resulting in similar (cross-domain) multi-agency initiatives being implemented and researched [26]. In Norway, FACT was implemented using a mandated team-level integration of primary and specialist MHC services from the outset. Teams undertake responsibility for clients, facilitate network collaboration with other network partners, and bridge service gaps, but fragmentation did hamper their functioning remained hampered by fragmentation [27,28]. In the city of Lille an integrated care network achieves flexible coordination of services by inter-organizational collaboration, requiring a strong value-driven sense of joint regional responsibility [25]. Given that both Norway, Lille, and FACT+DSS used different approaches to integrate services and address fragmentation, comparison highlights that addressing fragmentation does not require a single model, but rather context-sensitive alignment between team-level integration and network-level integration.

This study adds to the growing literature describing different initiatives aiming to enrich, adapt, or integrate FACT, reflecting its context-specific needs within the evolving care landscape. Often, deliberate model adaptations are needed for FACT to continue delivering recovery-oriented care, in line with its originally intended model principles [6]. Examples of other approaches enriching FACT include the use of resource groups [29], integration with a specialized provider of social services for SMI [30], or integration of specialized diagnosis-related treatment teams [31]. Additionally, these approaches aim to align care-delivery with six key principles defined for high-quality community-based MHC [32].

Several barriers and facilitators identified in this study are in accordance with findings from other implementation contexts, i.e. relating to the importance of leadership and shared vision [23,24,33], ownership and culture change [24,33], and the importance of establishing direct relationships between care providers [23,24].

### Implications & recommendations for policy

To improve coordination and integration of services, multi-agency collaboration between cross-domain partners is recognized as essential. The success and sustainability of such collaborations is strongly dependent on contextual factors, including shared leadership, collaborative governance, and funding. As pointed out by Van der Scheer (2023), as external incentives remain lacking, collaboration beyond institutional mandate often is heavily dependent on the personal motivation of its leaders [34]. This may introduce significant vulnerabilities upon change, for example, periodic elections at the municipal level may lead to changes in priorities and funding structures. The importance of context on integration of services and how this is characterized by horizontal complexity (i.e. regional/municipal level), vertical complexity (i.e. national level), and the local autonomy of actors, has been described in detail elsewhere [28].

International initiatives demonstrate that integration can be organized at different structural levels (i.e. within a team, or between network partners), each with distinct benefits and challenges. Challenges to team-level integration by mandate (e.g. Norway) may include being constrained by institutional boundaries or decentralized governance, whereas team-level integration without mandate (e.g. FACT+DSS) may require greater alignment due to separate mandates and funding structures. Alternatively, network-based approaches require strong coordination and commitment to avoid fragmentation or unclear accountability. As highlighted by Westen et al. (2025), integrating more services into a single FACT team may risk the team itself becoming fragmented from the network, emphasizing the importance of finding the optimal balance between team-level integration and network-level collaboration. Furthermore, as also illustrated in the findings of this study, just like organizing integrated care for clients, multi-agency collaborations at each structural level (e.g. within teams or between networks) will actively require continuous investment and development [6]. Using available insights, network partners must identify at which structural levels integration is most sustainable and contextually appropriate. Policy should facilitate network collaboration e.g. by incentivizing collaboration and shared responsibility, strengthening structural alignment between teams and networks, and reducing financial fragmentation.

A recognized barrier to network collaboration with FACT includes lacking understanding of mutual organizational and/or professional roles and responsibilities [16,18]. Our findings show that integration can also reshape professional and team role perceptions. FACT+DSS team members described experiencing shifts between roles relating to providing treatment vs. support and specialized vs. generalized care. For the team as a whole, integration with DSS-social workers allowed providing a more generalist, holistic approach to client care. Simultaneously, team members experienced an improved ability to practice their specialist role e.g. by having more direct patient time. These findings reflect a process of ‘re-professionalization’, where professionals redirect their time and expertise back to its core professional roles. This shift includes moving away from responsibilities allocated by necessity (e.g. outreach to care-avoiding individuals by social workers) or limited by system restrictions (e.g. medicalization of FACT limiting their community presence) [35]. Monitoring these dynamics over time could support care professionals to strengthen their expertise and optimize its efficient use.

Finally, our findings highlight the importance of the role of social work in promoting recovery, e.g. by advocating for clients’ needs and rights when navigating complex systems. It is important to monitor that the professional education for social work reflects the growing requirements and responsibilities associated with their professional roles and competencies (e.g. advocacy) [6].

### Recommendations for practice

This study also provides valuable insights for practice, supporting both current and future FACT+DSS teams to be implemented, as well as multi-agency initiatives in other contexts. Facilitators such as having a collaborative mindset, strong leadership, establishing equal importance between professionals, and developing direct relationships between team members, are essential for achieving and maintaining integration. To successfully sustain the desired level of integration, it is important to continue invest in these facilitators, including the allocation of necessary resources (i.e. time for period team meetings, coaching role), especially during periods of change within the team. Furthermore, interviews also revealed areas for future improvement; despite structural integration, integrated intake and shared treatment planning inclusive of DSS-social workers were not yet consistently embedded in daily practice. Here, continued stimulation of team members remains important, as well as reducing the impact of contributing factors (external stressors and lacking sufficient expertise).

### Limitations and further research

Several limitations should be acknowledged. First, lacking generalizability is inherent to qualitative research. Second, no formal FACT fidelity assessments using the FACTs-2017R model were available before and after integration. Third, clients could not be interviewed directly due to ethical constraints, although client representatives with lived FACT experience were included. Fourth, external perspectives (e.g. non-integrated teams, municipal partners, or public safety actors) were not included.

Future research could advance the continued development of network collaborations involving FACT, for example by examining the alignment of FACT+DSS teams with the FACTs-2017R fidelity framework, or by conducting in-depth comparative analyses of different multi-agency approaches. These studies could provide important insights on key factors to achieving an optimal balance between team-level integration and effective network collaboration. Valuable insights could be collected using (quantitative) measures regarding client (e.g. recovery, quality of life) outcomes, the number of care-avoiders and crisis incidents, client proportions receiving fully integrated treatment plans and intakes, and discharges to alternative providers. Additionally, broader societal benefits may be explored, including impact on public order and safety. Finally, more research is needed on the long-term effects, including costs and its potential redistribution of funds across the different financial streams and stakeholders.

## Conclusion

This study describes how participants experienced the integrated FACT+DSS approach to improve wide variety of themes relating to care-delivery, supporting recovery-oriented care for clients with SMI. Valuable lessons from its implementation can support future rollout of future initiatives and inform future policies and legislation.

## Additional File

The additional file for this article can be found as follows:

10.5334/ijic.9794.s1 Supplementary Materials. Tables S1 to S3.
