# Supplementary material for: The Integration of Flexible Assertive Community Treatment with District Social Support Service Teams for Patients with Severe Mental Illness in the Netherlands: A Qualitative Analysis of Team Members’ Experiences
Source: Int J Integr Care. 2026 Sep 8;26(3):16. doi: 10.5334/ijic.9794 (PMC13564374; doi:10.5334/ijic.9794)
Supplement: Supplementary file 1 [file ijic-26-3-9794-s1.pdf]

Supplementary materials

Table S1. Checklist of the Consolidated criteria for reporting qualitative studies (COREQ), 32-items.

No. Item	Guide questions/description	Reported on Page #
**Domain 1: Research team and reflexivity**		
Personal Characteristics		
1. Interviewer/facilitator	Which author/s conducted the interview or focus group?	Anne Kleijburg
2. Credentials	What were the researcher's credentials? E.g. PhD, MD	Msc.
3. Occupation	What was their occupation at the time of the study?	PhD student
4. Gender	Was the researcher male or female?	Female
5. Experience and training	What experience or training did the researcher have?	Related to qualitative research: Course during master's on "Advanced research methods" including qualitative research methods. Course "Interview to results" during PhD prior to interviews.<br>General: Bachelor and master's degrees in Biopharmaceutical sciences and Health Economics, Policy & Law
Relationship with participants		
6. Relationship established	Was a relationship established prior to study commencement?	During study visits the researcher was introduced to all team members, and more familiarized with some of the participants during walkalong days.
7. Participant knowledge of the interviewer	What did the participants know about the researcher? e.g. personal goals, reasons for doing the research	Academic background, current position within organization, field of study interest.
8. Interviewer characteristics	What characteristics were reported about the interviewer/facilitator? e.g. Bias, assumptions, reasons and interests in the research topic	None. There was no cause to assume researcher characteristics posed significant risks for bias or assumptions.

**Domain 2: study design**		
Theoretical framework		
9. Methodological orientation and Theory	What methodological orientation was stated to underpin the study? e.g. grounded theory, discourse analysis, ethnography, phenomenology, content analysis	Content analysis
Participant selection		
10. Sampling	How were participants selected? e.g. purposive, convenience, consecutive, snowball	Using (criterion-based) purposive sampling.<br><br>Both FACT+DSS teams were approached to participate in the interview study and requested to select at least 6 team members familiar with the working methods of the team, including at least 4 different specialisms, amongst which at least one social worker and a nurse specialist or psychiatrist. To include the client perspective in the interviews, two representatives from the client council from the MHC provider (GGZ Friesland) were requested to participate.
11. Method of approach	How were participants approached? e.g. face-to-face, telephone, mail, email	-Email to team leader with request to discuss amongst themselves who was willing to participate. Names were returned to book the interviews. The request did include specifications regarding the inclusion of at least 6 team members, preferably with as many different expertise/roles in this selection as feasible (e.g. nurses, psychologists, psychiatrist, social worker).<br>-Client council was contacted upon introduction via email by the MHC-provider representatives in steering group.<br>-Project manager was contacted directly via email.
12. Sample size	How many participants were in the study?	15, across a total of 12 interviews.
13. Non-participation	How many people refused to participate or dropped out? Reasons?	None directly refused, voluntary selection.<br>One interview was cancelled due to illness, and could not be rescheduled (changed employment).
Setting		
14. Setting of data collection	Where was the data collected? e.g. home, clinic, workplace	Workplace or using online Teams meeting (from workplace or home).
15. Presence of non-participants	Was anyone else present besides the participants and researchers?	No.
16. Description of sample	What are the important characteristics of the sample? e.g. demographic data, date	See table S2.
Data collection		

17. Interview guide	Were questions, prompts, guides provided by the authors? Was it pilot tested?	Questions were prepared using a semi-structured guide. When little response was provided or interesting topics were addressed additional follow-up questions could be asked.
18. Repeat interviews	Were repeat interviews carried out? If yes, how many?	No.
19. Audio/visual recording	Did the research use audio or visual recording to collect the data?	Audio recording.
20. Field notes	Were field notes made during and/or after the interview or focus group?	Yes.
21. Duration	What was the duration of the interviews?	Interview duration was planned to be circa 60 minutes. In practice, between 45 and 75 minutes, depending on the number of participants per interview (max 2, if preferred and mutually agreed upon), available time of the participant, and the elaborateness of their responses.
22. Data saturation	Was data saturation discussed?	Not extensively based on data alone.<br><br>Sample size for sufficient saturation was estimated based on information power and researcher perspective based on earlier experiences/observations. First, the minimum of 6 participants per team was assumed to be a sufficient minimum considering the sampling criteria and high level of information power. Second, although not being formal participants for data collection, during several on-site walkalongs by the (first author) researcher, experiences of other team members were also discussed, which did not provide indication to insufficient saturation. Third, the (first author) researcher participated in each periodic (6 to 8 week) steering group meetings, to remain informed about the implementation process and team's current situation.
23. Transcripts returned	Were transcripts returned to participants for comment and/or correction?	Full transcripts were not returned to the participants. Participants were informed regarding the purpose of the study and planned dissemination prior to the interview (via email and verbally at the start of the interview). When clear participants were asked for consent to include citations from the interviews in the final manuscript, and their desire to approve any citations before use.
**Domain 3: analysis and findings**		
Data analysis		
24. Number of data coders	How many data coders coded the data?	Two.
25. Description of the coding tree	Did authors provide a description of the coding	Not included. Upon request.

	tree?	
26. Derivation of themes	Were themes identified in advance or derived from the data?	Both. Using the framework and interview questions first themes were identified but during analysis additional themes were added.
27. Software	What software, if applicable, was used to manage the data?	MAXQDA
28. Participant checking	Did participants provide feedback on the findings?	Three co-authors of the stakeholder organizations were included, of which two participated in the interviews. Both co-auteurs have been involved prior to and throughout the entire implementation period/study, including an active team member to ensure correct interpretation of results.
Reporting		
29. Quotations presented	Were participant quotations presented to illustrate the themes/findings? Was each quotation identified? e.g. participant number	Yes. No participant number was provided, only their job title.
30. Data and findings consistent	Was there consistency between the data presented and the findings?	Yes.
31. Clarity of major themes	Were major themes clearly presented in the findings?	Yes.
32. Clarity of minor themes	Is there a description of diverse cases or discussion of minor themes?	No minor themes were explicitly discussed.

Table S2. Characteristics of interviewed team members, client representatives and project leaders at time of interview.

Respondent	Team	Job title	Organization of employment	Years in current job function	Years of total experience
**R1**	Urban	Psychiatrist & coach	Mental healthcare provider	6y	30+
**R2**	Urban	Social psychiatric nurse	Mental healthcare provider	2y	5+
**R3**	Urban	Social psychiatric nurse	Mental healthcare provider	1y	5+
**R4**	Urban	Nurse	Mental healthcare provider	2y	10+
**R5**	Urban	Nurse	Mental healthcare provider	4y	5+
**R6**	Urban	Social worker	Amarylis (provider in municipality Leeuwarden)	8y	10+
**R7**	Rural	Psychiatrist	Mental healthcare provider	3y	10
**R8**	Rural	Nurse practitioner	Addiction healthcare provider	8y	10+
**R9**	Rural	Ambulatory nurse	Mental healthcare provider	21y	20+

**R10**	Rural	Social psychiatric nurse	Mental healthcare provider	15y	20+
**R11**	Rural	Nurse	Addiction healthcare provider	3y	30+
**R12**	Rural	Social worker	Municipality Súdwest-Fryslân	1y	30+
**R13**	n.a.	Client representative	Mental healthcare provider	5y	n.a.
**R14**	n.a.	Client representative	Mental healthcare provider	4y	n.a.
**R15**	n.a.	External project leader, primary responsible consultant.	n.a.	3y	5+

Table S3. Overview of the interview topics and questions per Saunders et al. framework element.

**Framework element**	**Topic**	**Interview question**
	General introduction	Short introduction, work experience, time & role in team
**Fidelity**	Perceived pilot aims	Can you describe in your own words what the most important aims of the integration were for your team?
**Dose received & delivered**	Experiences start integration & implementation materials/resources	Can you describe how your team prepared for implementation and how you experienced the initial phase?<br><br>Were there sufficient supportive materials?<br><br>What could have gone better/would you change now?
**Dose received & delivered**	Experiences towards middle/end phase of pilot	Did you recognize/experience a transition between the initial phase and a sense of full implementation/being completely adjusted to integration?<br>How long did this take? Based on what did you notice this?
**Dose delivered**	(Impact on) Daily practices	Can you describe your current daily routine/practices?<br>How do these differ from before integration? What changes here do you consider most important/meaningful?
**Dose delivered**	Involvement and access to professionals from different expertise's/stakeholders	Given that the integration consists of collaboration with social workers, addiction care specialists and occupational consultants; how do you experience the level of access/availability and involvement/collaboration with each of the expertise's?<br>For what percentage of the patients/treatment plans do you estimate to collaborate with each?<br>What do you think still could be improved here and why?
**Reach**	Impact on caseload mix/size and care-avoiders	Do you think integration has impacted how the target population is defined? And the case-mix?<br>Do you notice difference in the clients being reached now that were not reached before?
**Recruitment**	Impact on enrolment and discharge	Did you notice any changes in how new clients are enrolled into the team? What are these changes and how do you think this was impacted?<br><br>Did you notice any changes in how current clients are discharged from the team? What are these changes and how do you think this was impacted?
**Dose received**	Perceptions on most important improvements and benefits of integration	What do you consider to be the most important improvements or benefits from the integrated approach for clients? And for team members? Did you receive feedback from clients?<br>Do you think there are also downsides/negatives to this new approach?

**Fidelity**	Perceptions on achieving integration goals	Do you believe all the aims established prior to integration are achieved? What could still be improved? Other additions needed?
**Context**	Perceived facilitators	What did you experience to be important facilitators for success during implementation and now?
**Context**	Perceived barriers	What did you experience to be important barriers to success during implementation and now?
**Closing statements**	Final messages	Before ending the interview, are there any last things you would like to share? Feelings, experiences, recommendations, feedback etc
